# Transcriptome Analysis of Genes Involved in Dendrobine Biosynthesis in *Dendrobium nobile* Lindl. Infected with Mycorrhizal Fungus MF23 (*Mycena* sp.)

**DOI:** 10.1038/s41598-017-00445-9

**Published:** 2017-03-22

**Authors:** Qing Li, Gang Ding, Biao Li, Shun-Xing Guo

**Affiliations:** Institute of Medicinal Plant Development, Peking Union Medical College, Chinese Academy of Medical Sciences, Beijing, 100193 People’s Republic of China

## Abstract

Content determination and microscopic observation proved that dendrobine accumulation in the stem of *Dendrobium nobile* Lindl. increased after infection with mycorrhizal fungus MF23 (*Mycena* sp.). Large-scale transcriptome sequencing of symbiotic and asymbiotic *D. nobile* revealed that 30 unigenes encoding proteins were possibly related to the biosynthesis of dendrobine sesquiterpene backbone. A qRT-PCR experiment of 16 unigenes, selected randomly, proved that there were significant changes in the expression levels of *AACT*, *MVD*, *PMK* and *TPS21* at 9 weeks after inoculation. These results implied that MF23 might stimulate dendrobine biosynthesis by regulating the expressions of genes involved in the mevalonate (MVA) pathway. The biogenetic pathway of dendrobine was suggested systematically according to the structural features of dendrobine alkaloids and their sesquiterpene precursors, which implied that post-modification enzymes might play a major role in dendrobine biosynthesis. Thus, genes encoding post-modification enzymes, including cytochrome P450, aminotransferase and methyltransferase, were screened for their possible involvement in dendrobine biosynthesis. This study provides a good example of endophytes promoting the formation of bioactive compounds in their host and paves the way for further investigation of the dendrobine biosynthetic pathway.

## Introduction

The stem of *Dendrobium nobile* Lindl. (Orchidaceae) has been used in Traditional Chinese Medicine (TCM) and as an herbal medicine in many Asian countries for hundreds of years with special pharmacological effects on gastritis, diabetes, cancer and ageing^1–4^. It has been officially listed as “Shi Hu” in different versions of the Pharmacopoeia of the People’s Republic of China. However, due to the destruction of wild environments and the increasing market demand, wild *D. nobile* (listed on the National Key Protected Wild Medicinal Plants) is being increasingly depleted.

The major bioactive components of *D. nobile* include alkaloids, polysaccharides, and polyphenols^5–7^. Among these compounds, sesquiterpene alkaloid dendrobine^8^ has been regarded as the quality standard of *D. nobile*. Modern pharmacology has demonstrated that this group of alkaloids has remarkable anti-hypertensive, anti-cancer, analgesic, and antipyretic effects in clinical studies^9^. Dendrobine is primarily obtained by extraction^10, 11^ or chemical synthesis^12, 13^; however, neither method is efficient enough because of the low content in *D. nobile* ^14^ and technical problems in total synthesis. The high market demand has led to excessive harvesting and exploitation of *D. nobile*, which has contributed to resource depletion. Considering the advantage of biotechnology and successful examples applied in *D. nobile* tissue culture, their use for increasing the dendrobine content of *D. nobile* in large quantities *in vitro* is becoming possible to not only meet market demand but also protect the wild resource of *D. nobile*.

Mycorrhizal fungi play a critical role in the lifetime of orchids; research has suggested that these fungi can provide inorganic and organic nutrients for seed germination and early seedling development^15, 16^. The most direct evidence indicated that mycorrhizal orchids could acquire more P, N and water than those of non-mycorrhizal controls. Thus far, numerous mycorrhizal fungi with growth-promoting effects have been isolated from the roots of wild *Dendrobium officinale* Kimura et Migo and D. *nobile* in our laboratory^17–19^. Some fungi have been used in actual production to enhance the growth of *D. officinale* and *D. nobile*. Our previous reports showed that when *D. nobile* was inoculated with MF23 (*Mycena* sp.), a mycorrhizal fungus isolated from the roots of *D. officinale*, its total alkaloid content significantly increased (18.3%)^20, 21^, indicating that MF23 might be able to promote dendrobine biosynthesis. However, the relationship between mycorrhizal fungi and their hosts is still not clear, which hinders the application of mycorrhizal fungi. Thus, understanding the regulatory mechanism of MF23 and improving the dendrobine content of *D. nobile* are of great importance.

The development of molecular biology has prompted the use of RNA-seq, a powerful tool in novel gene discovery and prediction. The advances in next-generation sequencing technologies, such as 454 pyrosequencing, have made sequencing cheaper and faster, and obtaining large datasets has become a viable option. Thus far, RNA-seq has been successfully applied to study the regulation of gene expression for many plants, including *D. offcinale* ^22–24^.

In this study, we collected and analysed the transcriptome data for the stems of asymbiotic (control group) and symbiotic *D. nobile* (model group) using RNA-seq technology based on an Illumina HiSeq 4000 platform. qRT-PCR experiments involving key genes, including *AACT*, *MVD*, *PMK* and *TPS21*, related to the sesquiterpene skeleton formation of dendrobine were performed. These experiments provided an overview of the *D. nobile* stem transcriptome and candidate genes encoding enzymes involved in the dendrobine biosynthetic pathway. The biogenetic pathway of dendrobine was systematically postulated according to the structural features of dendrobine alkaloid analogues and sesquiterpene precursors. qRT-PCR evaluations of genes encoding cytochrome P450, aminotransferase and methyltransferase suggested that post-modification enzymes might play vital roles in the biogenetic pathway of dendrobine alkaloid. The results in this report provide a good example of endophytes promoting the formation of bioactive compounds in their host and pave the way for further investigations of the dendrobine biosynthetic pathway.

## Results

### Morphological characteristics of *D. nobile*

**Figure 1:**
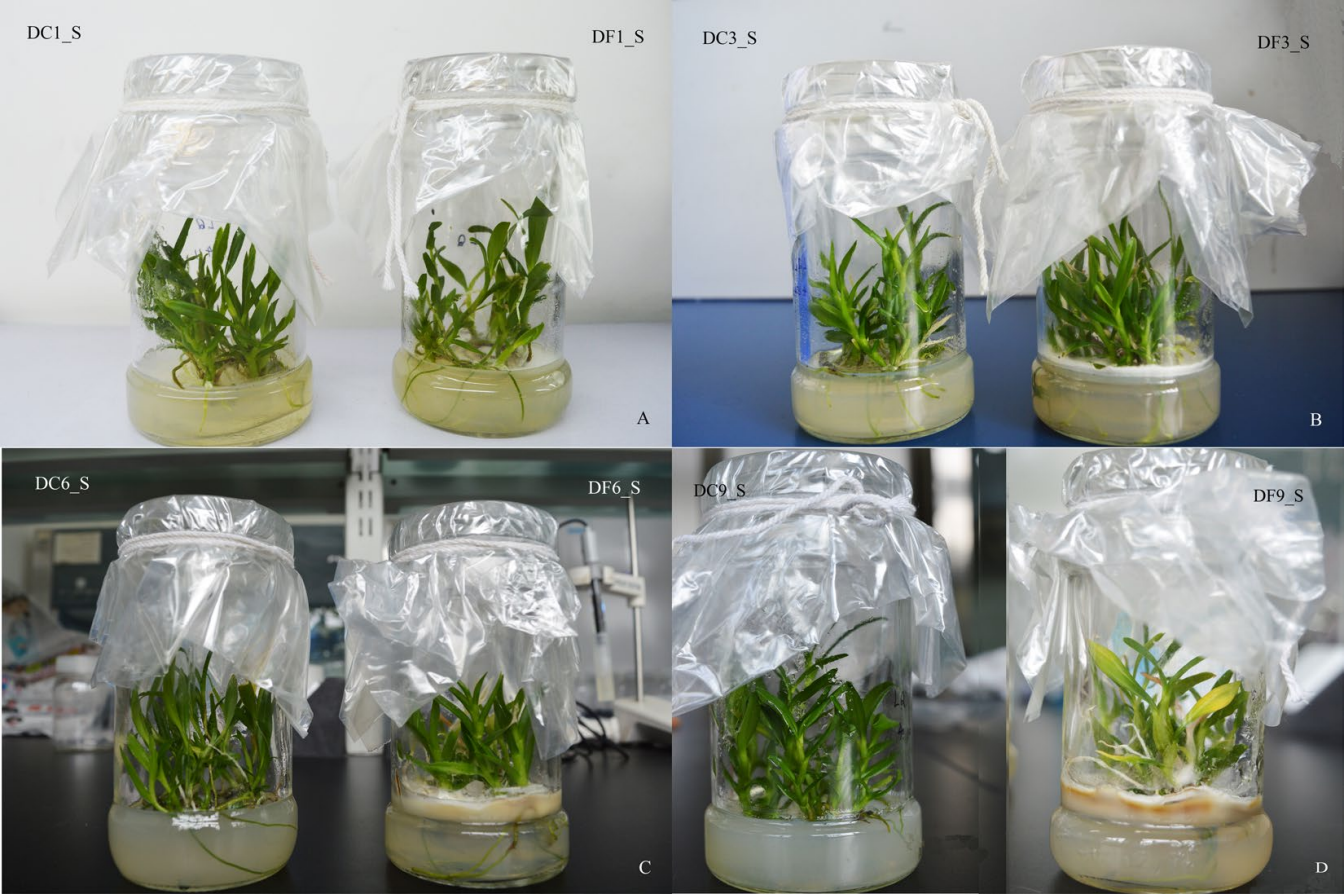
The effect of MF23 on the growth of *D. nobile* tissue culture seedlings (**A**,**B**). (**A**) *D. nobile* 1 week after inoculation (DF1_S) and the control (DC1_S). (**B**) *D. nobile* 3 weeks after inoculation (DF3_S) and the control (DC3_S). (**C**) *D. nobile* 6 weeks after inoculation (DF6_S) and the control (DC6_S). (**D**) *D. nobile* 9 weeks after inoculation (DF9_S) and the control (DC9_S).

A comparison of the differences in morphological indexes between the model and control groups (Supplementary Fig. 1) revealed significant increases in the stem diameter (57.69%) and drying rate (35.77%) in the model group 9 weeks after inoculation. However, Fig. 1 shows that the leaves of symbiotic *D. nobile* turned yellow at the same time, suggesting that *D. nobile* was stressed by MF23 in some way.

### Changes in dendrobine contents in *D. nobile* stems

**Figure 2:**
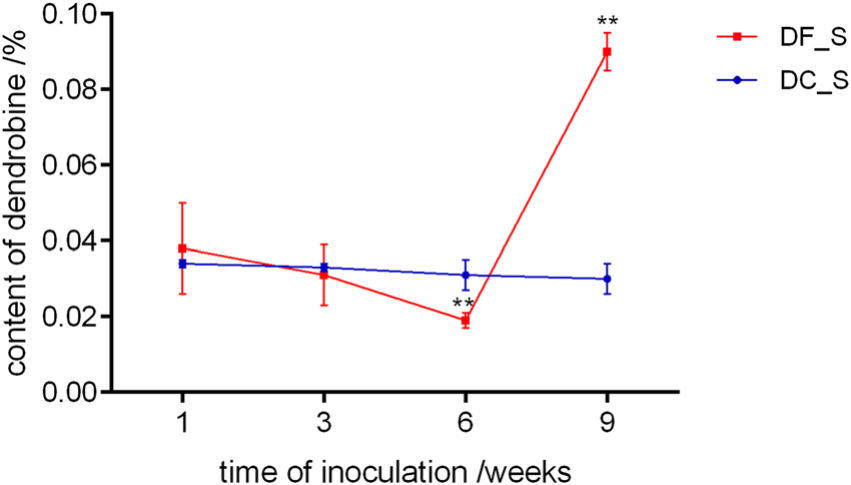
The effect of MF23 on the dendrobine content in *D. nobile* tissue culture seedlings. Values are presented as the means ± SD (n = 3); values with ** are of statistical significance at *P* < 0.01.

Gas chromatography (GC) analysis was used to evaluate the dendrobine contents in *D. nobile* stems at 1, 3, 6, 9 weeks after inoculation and in the control. Dendrobine accumulation in samples is shown in Fig. 2. The changes in dendrobine content occurred at 6 and 9 weeks in model group (P < 0.01). At 6 weeks, the content of dendrobine was 38.7% less than that in the control but was as much as 3-fold higher than that of the control at 9 weeks, implying that the effect of MF23 in stimulating the biosynthesis of dendrobine was formed in the later period of symbiotic culture.

### MF23-*D. nobile* root associations

**Figure 3:**
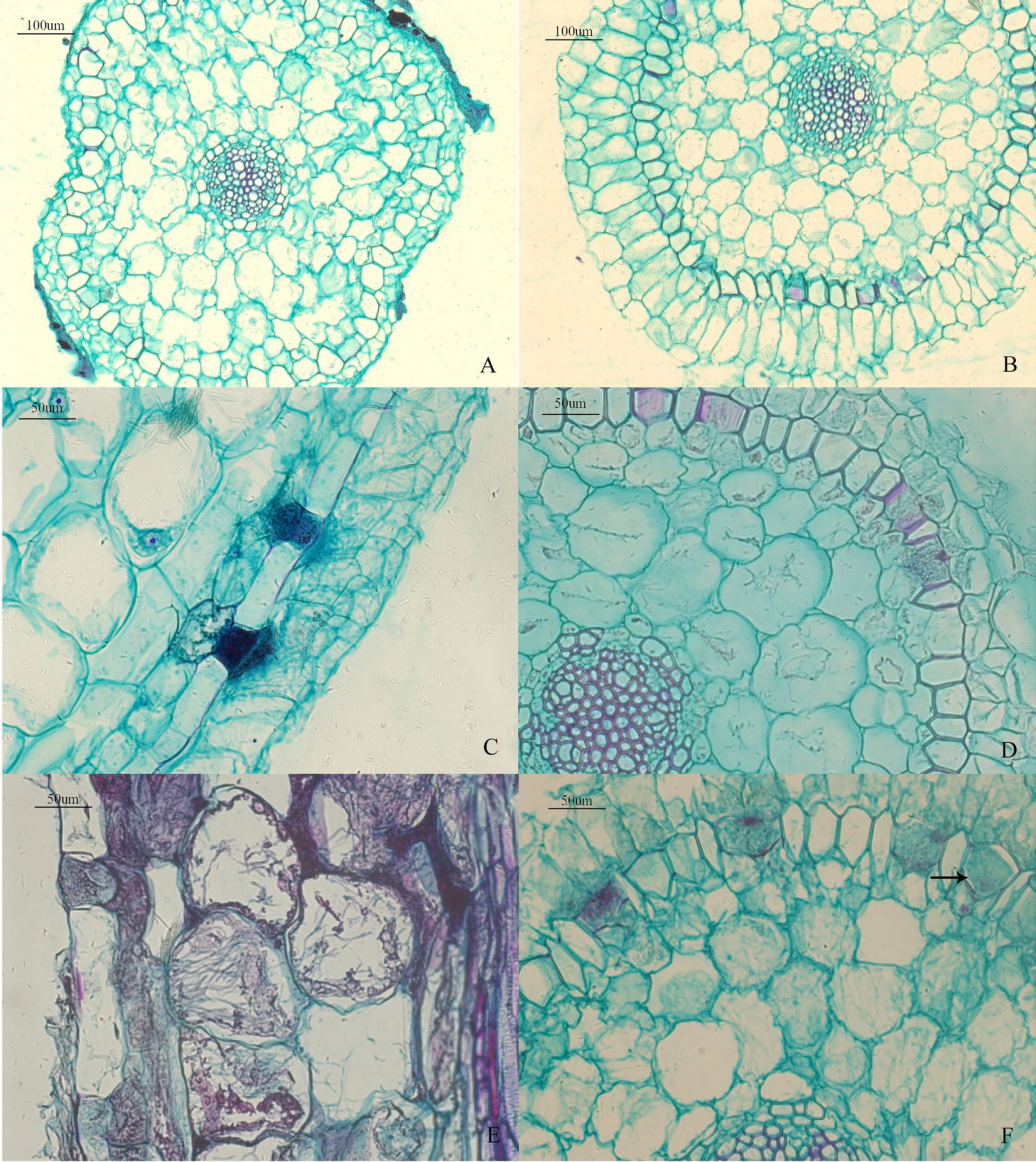
Light micrographs of interactions between the roots of *D. nobile* and MF23 (**A–F**). (**A**) Transverse section of the control (×10). (**B**) Transverse section of the root after 1 week of symbiotic cultivation (×10). (**C**) Vertical section of the root after 3 weeks of symbiotic cultivation (×20). Distribution of a large number of hyphae outside the passage cells of the exodermis. (**D**) Transverse section of the root after 6 weeks of symbiotic cultivation (×20). The hyphae penetrated the cortex cell walls and colonized neighbouring cells through passage cells into the cortical region. (**E**) Vertical sections of the root after 6 weeks of symbiotic cultivation (×20). Hyphae passed through the cell wall to progress towards the other cortical cells. (**F**) Transverse section of the root after 9 weeks of symbiotic cultivation (×20). The peloton was formed in the passage cells.

After 3 weeks of symbiotic cultivation, light microscopy of paraffin sections of *D. nobile* roots revealed that the hyphae of MF23 penetrated the epidermal cells of the host roots and spread from cell to cell. A mass of hyphae also gathered outside the passage cells of the exodermis (Fig. 3C). After 6 weeks, the hyphae penetrated the cortex cell walls and colonized neighbouring cells through the passage cells into the cortical region. The hyphae thrived in the cortical cells that were adjacent to the exodermis and penetrated the host’s cell walls to progress towards the other cortical cells. The connections between the hyphae in adjacent cells through the cell wall were noteworthy (Fig. 3D,E). After 9 weeks, we observed the distribution of a large number of hyphae in the cortical cells of the *D. nobile* roots. In this study, since symbiotic cultivation time was not long enough, peloton, a typical structure of orchid mycorrhizae, was not found in the cortical region; it appeared only in the passage cells. However, the tendency of typical orchid mycorrhizae formation was still shown by the gathering of hyphae in some cortical cells (Fig. 3F). In contrast, light microscopic analysis of the root’s paraffin section revealed that the samples cultured with MF23 for 1 week, and the control, remained uncolonized (Fig. 3A,B).

### Sequencing and *de novo* transcriptome assembly

To obtain the *D. nobile* transcriptome expression profile upon MF23 infection, one non-normalized library was constructed using *D. nobile* stems infected with MF23, with normal *D. nobile* stems as a control. In this study, all samples were sequenced in two biological replicates. In total, 430,149,656 Illumina paired-end (PE) raw reads were generated (Supplementary Table S1). The raw reads are available in the NCBI SRA database under the accession number PRJNA338366. After removing adaptor sequences, ambiguous nucleotides, and low-quality sequences, 414,890,782 clean reads remained. Assembly of clean reads yielded 207,283 unigenes in the range of 201–17,019 bp with an N50 length of 857 bp (Supplementary Fig. S2).

### Sequence annotation

After eliminating repeated and short-length sequences, 207,283 non-redundant unigenes were presented to 7 public databases (Supplementary Table S2) for similarity searches. Analyses showed that 56,378 unigenes (27.19%) had significant matches in the Nr database, 34,493 (16.64%) in the Nt database, and 58,431 (23.36%) in the Swiss-Prot database. In total, there were 83,778 unigenes (40.41%) successfully annotated in at least one of these 7 public databases, and 8015 unigenes (3.86%) could be annotated in all databases.

**Figure 4:**
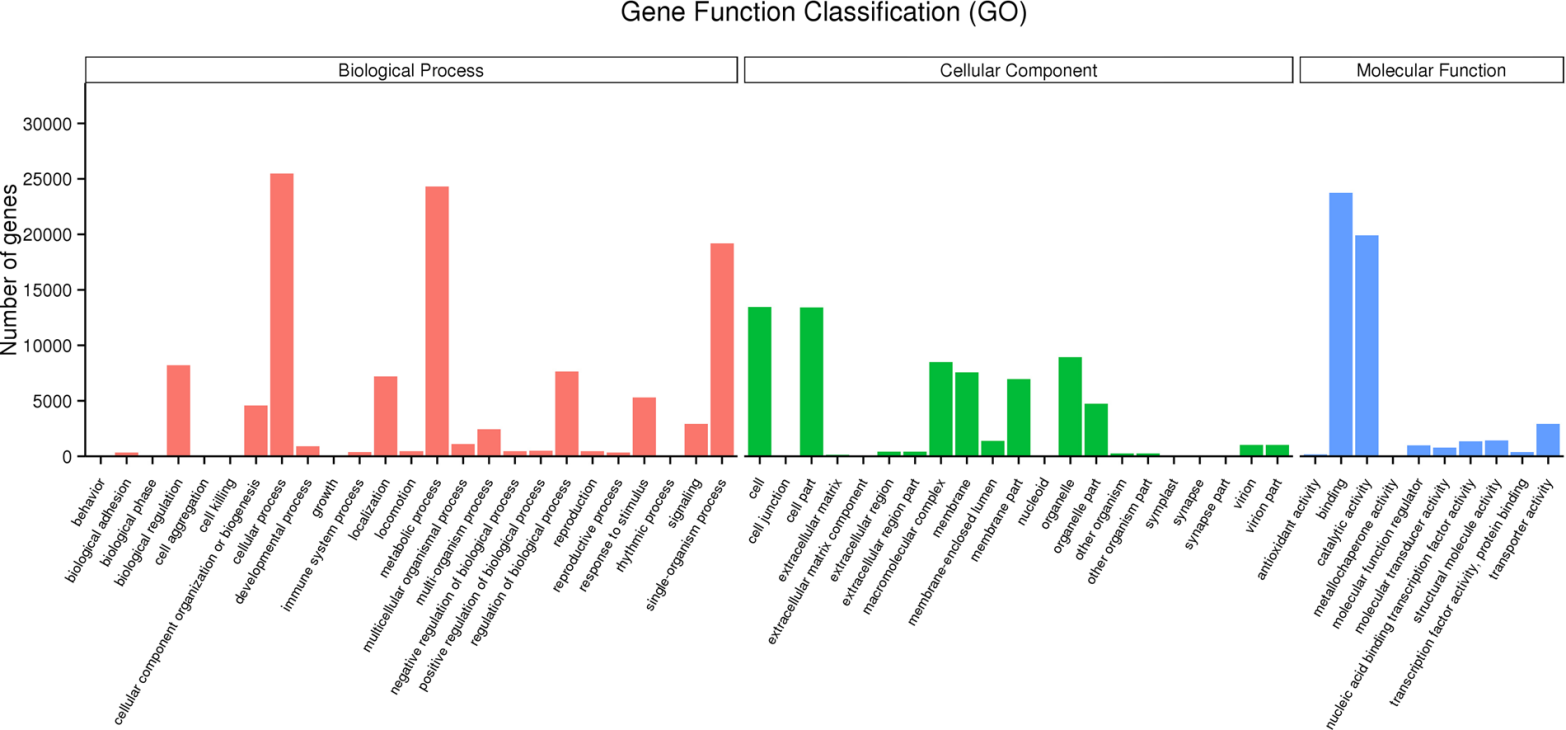
GO categorization of non-redundant unigenes.

According to Gene Ontology (GO), an international standardized gene functional classification system, 47,372 non-redundant unigenes were classified into three major functional ontologies (biological process, cellular component and molecular function) (Fig. 4). For biological process (BP), the dominant subcategories were ‘cellular process’ (25,487) and ‘metabolic process’ (24,304). In the category of cellular component (CC), ‘cell’ (13,442), ‘cell part’ (13,434) and ‘organelle’ (8,915) were highly represented. Among molecular function (MF) terms, ‘binding’ (23,731) and ‘catalytic activity’ (19,927) were the most represented. However, within each of these three categories, there were a few genes assigned to subcategories of ‘cell aggregation’, ‘symplast’ and ‘metallochaperone activity’.

**Figure 5:**
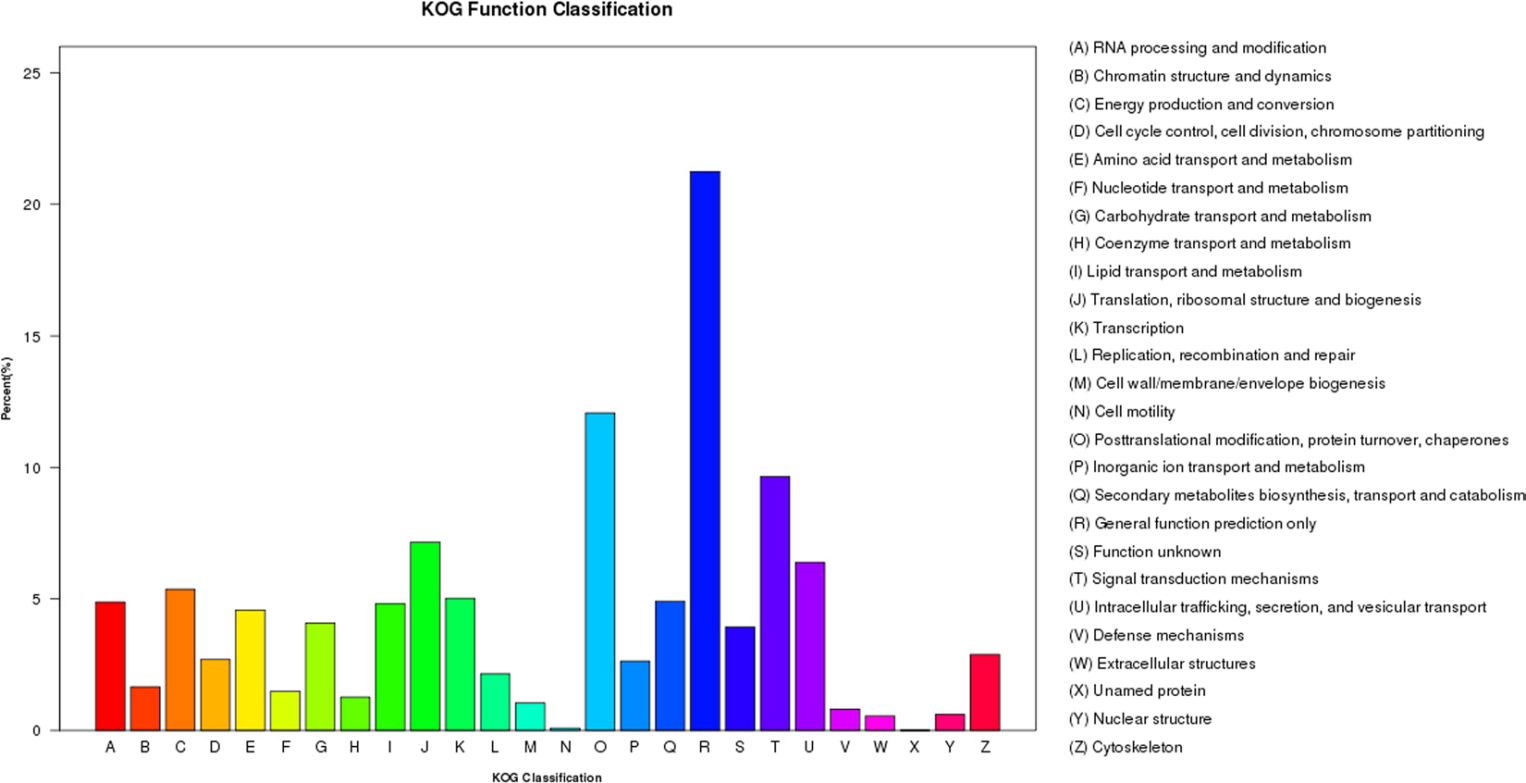
KOG annotation of putative proteins.

In addition, all unigenes were subjected to a search against the Eukaryotic Orthologous Groups of Proteins (KOG) database for functional prediction and classification. We subdivided 27,087 non-redundant unigenes into 26 KOG classifications (Fig. 5). Among them, the cluster of ‘general function prediction only’ (5,755) was the largest group, followed by ‘post-translational modification, protein turnover, chaperon’ (3,269), ‘signal transduction mechanisms’ (2,613), ‘translation, ribosomal structure and biogenesis’ (1,939) and ‘intracellular trafficking, secretion, and vesicular transport’ (1,732). Only a few unigenes were assigned to ‘cell motility’ (24) and ‘unnamed protein’ (1).

**Figure 6:**
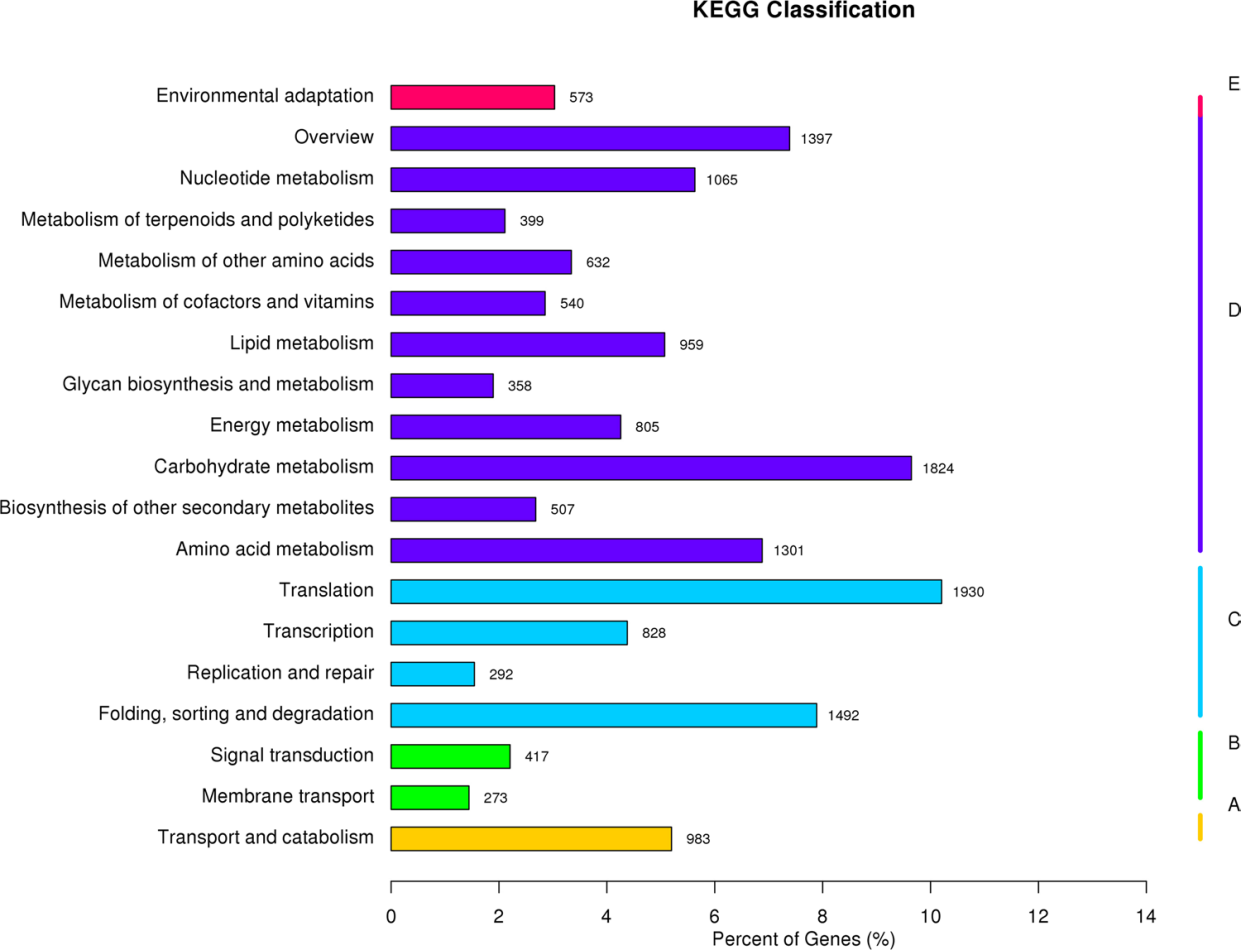
KEGG annotation of putative proteins.

Unigene metabolic pathway analysis was also conducted using the Kyoto Encyclopaedia of Genes and Genomes (KEGG) annotation system. According to KEGG, 18,911 unigenes were assigned to 131 pathways (Fig. 6). The pathways involved in the largest number of unique transcripts were ‘translation’ (1,930), followed by ‘carbohydrate metabolism’ (1,827).‘Membrane transport’ (273) was the smallest group.

### Identification of differentially expressed genes (DEGs)

**Figure 7:**
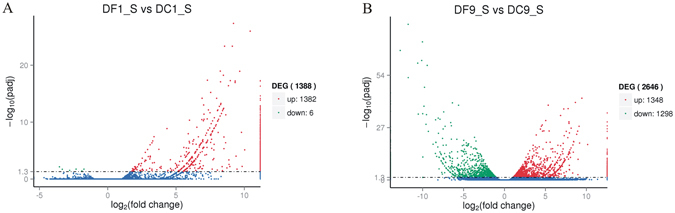
Volcano plots displaying differentially expressed genes between symbiotic and asymbiotic samples. (**A**) DF1_S compared with DC1_S. (**B**) DF9_S compared with DC9_S. The y-axes correspond to the mean expression values of log 10 (p-value), and the x-axes display the log2 fold change values. The red dots represent the up-regulated expressed transcripts (P < 0.05, false discovery rate (FDR) q < 0.05) between DF_S and DC_S; the green dots represent the transcripts whose expression levels were down-regulated (P < 0.05, FDR q < 0.05).

Differential expression analysis was first performed between the two treatments. DEGs (q-value < 0.005 and |log2 (fold change)| > 1) were defined as genes that were significantly enriched or depleted in one treatment relative to the other one. In this study, 1,388 DEGs were observed between DF1_S and DC1_S (1,382 up-regulated, 6 down-regulated). Moreover, between DF9_S and DC9_S, 2,646 genes were expressed at significantly different levels (1,348 up-regulated, 1,298 down-regulated) (Fig. 7).

### Functional categorization of the DEGs

**Figure 8:**
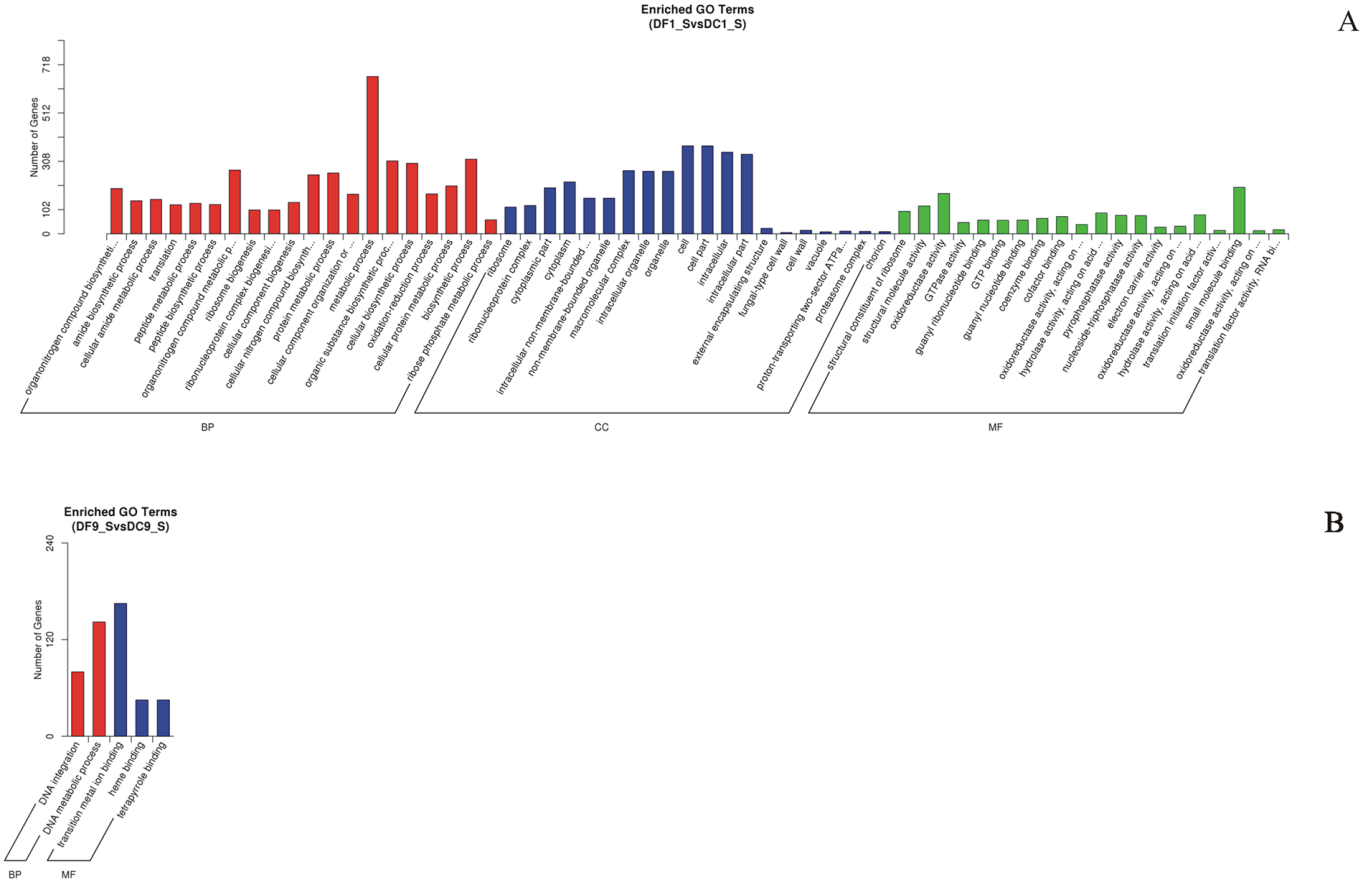
GO functional classification of differentially expressed genes. (**A**) DF1_S *vs.* DC1_S, (**B**) DF9_S *vs.* DC9_S.

Among all DEGs, there were 175 and 5 enriched GO terms related to various biological processes for the DF1_S *vs.* DC1_S and DF9_S *vs.* DC9_S, respectively (Fig. 8). For DF1_S *vs.* DC1_S, many of the enriched terms were involved in cellular component and metabolism, especially the metabolism of nitrogen compounds. These terms included ‘cell’ (GO:0005623), ‘cell part’ (GO:0044464), ‘metabolic process’ (GO:0008152), ‘biosynthetic process’ (GO:0009058), ‘organonitrogen compound metabolic process’ (GO:1901564) and ‘cellular nitrogen compound biosynthetic process’ (GO:0044271). These findings were consistent with the results from the morphology (a 57.69% increase in stem diameter) and metabolome analyses (a threefold increase in dendrobine content). However, for DF9_S *vs.* DC9_S, the numbers of unigenes associated with DNA metabolic and substance binding were quite high, very different from those of DF1_S *vs.* DC1_S, implying that MF23 influenced *D. nobile* in different biological processes at different stages.

KEGG pathway analysis of DEGs from DF1_S *vs.* DC1_S and DF9_S *vs.* DC9_S revealed 113 and 125 pathways, respectively. The top 20 pathways of DF1_S *vs.* DC1_S were mainly involved in amino acid metabolism and respiratory metabolism, indicating that MF23 sped up the metabolism of *D. nobile* at an early stage. In contrast, in DF9_S *vs.* DC9_S, the pathway of ‘purine metabolism’ was involved in the largest number of unique transcripts, followed by ones related to ‘phenylpropanoid biosynthesis’. Moreover, unigenes associated with plant-pathogen interactions and biosynthesis of defensive substances such as flavonoids, isoflavones and alkaloids were relatively high in number, suggesting that MF23 might activate the defensive mechanism of *D. nobile* (Table 1).

**Table 1:** Summary of the top 20 KEGG pathways in DEGs.

#	KEGG pathway	Number of unigenes	Percentage (%)
DF1_S *vs*. DC1_S			
1	Ribosome	111	12.82
2	Citrate cycle (TCA cycle)	25	2.89
3	Two-component system	10	1.15
4	Carbon metabolism	55	6.35
5	Phagosome	22	2.54
6	Oxidative phosphorylation	29	3.35
7	Carbon fixation in photosynthetic organisms	16	1.85
8	Pentose phosphate pathway	13	1.50
9	Glyoxylate and dicarboxylate metabolism	17	1.96
10	Biosynthesis of amino acids	38	4.39
11	2-Oxocarboxylic acid metabolism	14	1.62
12	RNA transport	27	3.12
13	Synthesis and degradation of ketone bodies	3	0.35
14	Glycolysis/Gluconeogenesis	20	2.31
15	C5-Branched dibasic acid metabolism	2	0.23
16	Protein processing in endoplasmic reticulum	30	3.46
17	Pyruvate metabolism	16	1.85
18	Proteasome	13	1.50
19	Alanine, aspartate and glutamate metabolism	11	1.27
20	Benzoate degradation	3	0.35
DF9_S *vs.* DC9_S			
1	Phenylpropanoid biosynthesis	26	4.63
2	Phenylalanine metabolism	18	3.20
3	Starch and sucrose metabolism	21	3.74
4	Flavonoid biosynthesis	8	1.42
5	Tropane, piperidine and pyridine alkaloid biosynthesis	6	1.07
6	Purine metabolism	37	6.58
7	Plant hormone signal transduction	14	2.49
8	Cyanoamino acid metabolism	8	1.42
9	Mineral absorption	4	0.71
10	Protein processing in endoplasmic reticulum	23	4.09
11	Stilbenoid, diarylheptanoid and gingerol biosynthesis	6	1.07
12	Isoquinoline alkaloid biosynthesis	5	0.89
13	Carotenoid biosynthesis	5	0.89
14	Tyrosine metabolism	7	1.25
15	Selenocompound metabolism	5	0.89
16	Plant-pathogen interaction	14	2.49
17	Linoleic acid metabolism	3	0.53
18	Folate biosynthesis	4	0.71
19	Phenylalanine, tyrosine and tryptophan biosynthesis	5	0.89
20	Carbon fixation in photosynthetic organisms	8	1.42

### Unigenes involved in backbone formation of sesquiterpene alkaloid dendrobine biosynthesis

**Figure 9:**
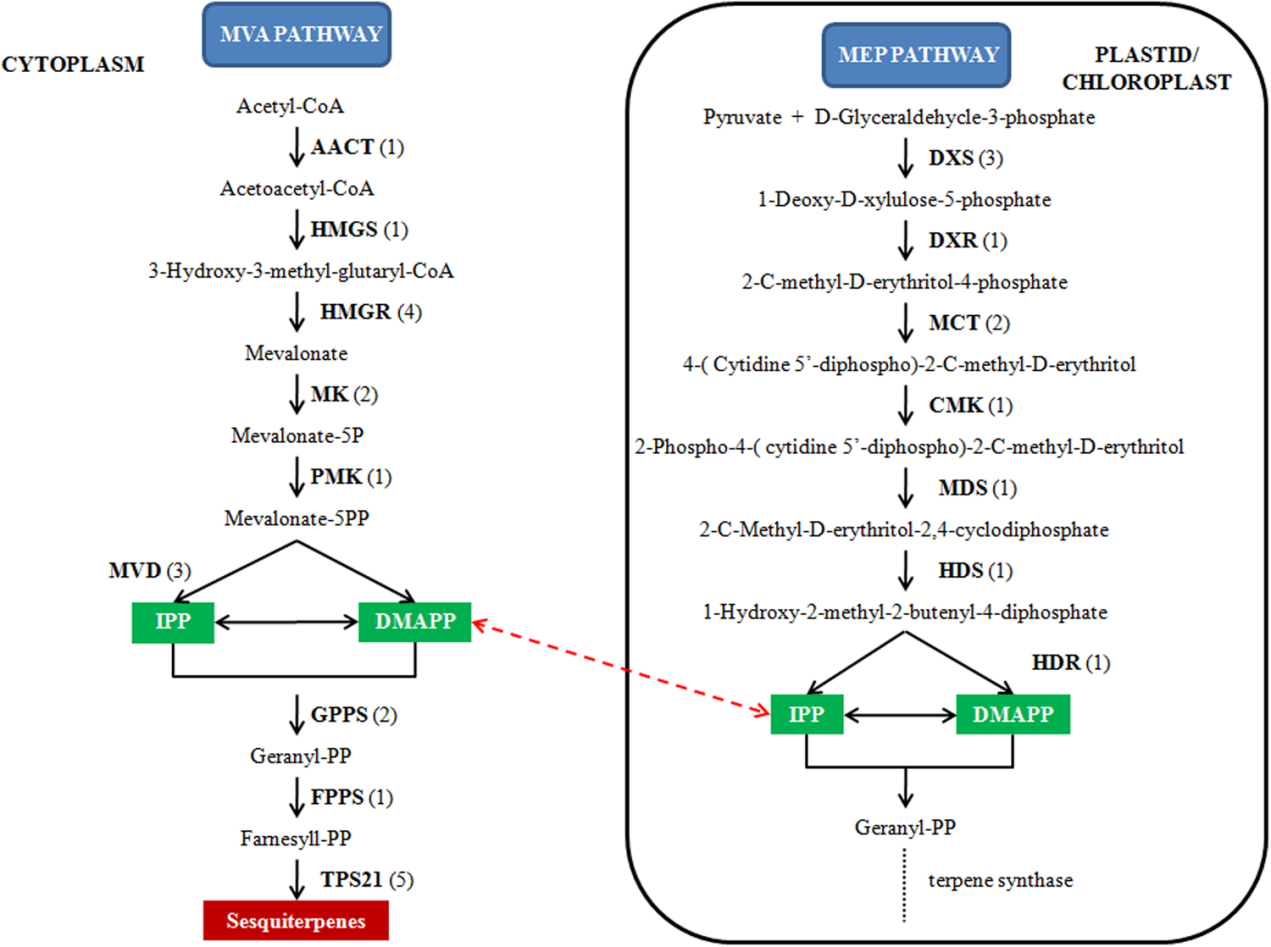
Putative genes involved in the MVA and MEP pathways. The numbers in brackets following each gene name indicate the number of unigenes annotated to that gene. For each gene abbreviation, its full name and other information can be verified from Supplementary Table S3.

Several reports and the structural features of dendrobine suggest its sesquiterpene origin^25, 26^. Evidence has shown that the upstream biosynthetic pathways involved in sesquiterpene backbone construction are conserved (Supplementary Figs S3 and S4). Although the construction of the sesquiterpene backbone of dendrobine was verified to be involved in the mevalonate (MVA) pathway^27^, there was no evidence to support the idea that dendrobine biosynthesis did not originate from the methyl-D-erythritol 4-phosphate (MEP) pathways. Thus, the unigenes encoding the key enzymes in these two pathways (MVA and MEP) were identified by searching the names of the annotated genes. As a result, 16 types key enzyme genes (Fig. 9) involved in terpenoid backbone biosynthesis were mined.

### Validation and expression analysis of genes involved in the formation of the backbone of the sesquiterpene alkaloid dendrobine

**Figure 10:**
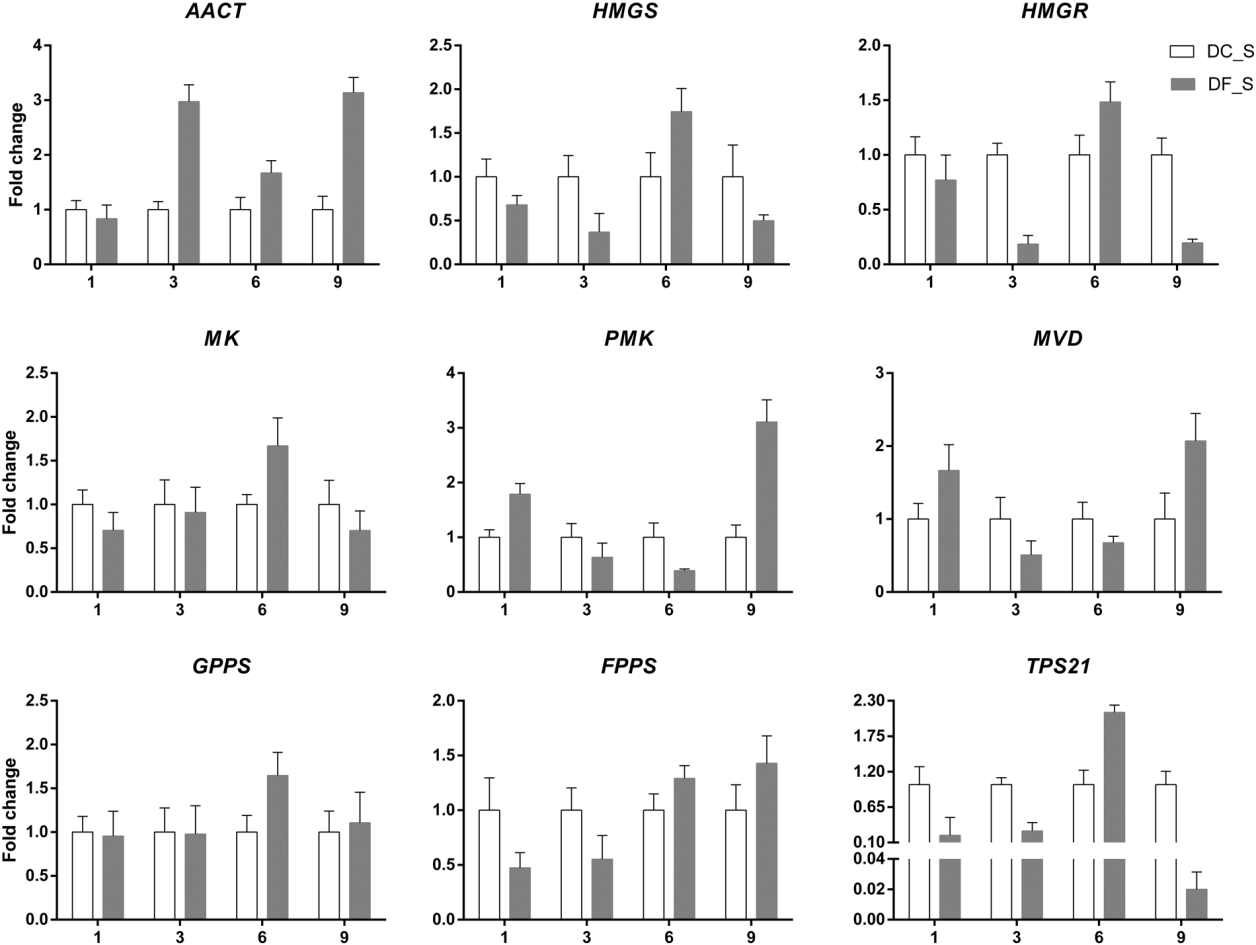
qRT-PCR analysis of key enzyme-coding genes involved in the MVA pathway in *D. nobile*. The y-axes correspond to the mean fold changes in expression values, and the x-axes display the times of symbiotic culture (/weeks). White bars represent the control group (DC_S), and grey bars represent the model group (DF_S). For each qRT-PCR validation, three technical replicates were used, with a minimum of three biological replicates.

In this study, 16 key enzyme-coding genes associated with the formation of the backbone of the sesquiterpene alkaloid dendrobine were chosen for qRT-PCR analysis. The expression levels of both *PMK* and *MVD*, involved in MVA pathway (Fig. 10), were up-regulated at 9 weeks after inoculation, when the dendrobine content was high. The dendrobine content decreased at 6 weeks after inoculation, corresponding to the time when the expression levels of both the *PMK* and *MVD* genes were down-regulated, implying a positive correlation between dendrobine biosynthesis and *PMK* and *MVD* expression. Interestingly, the expression of *TPS21*, which catalyses the formation of sesquiterpenes from farnesyl pyrophosphate (FPP), was significantly up-regulated (2.12-fold) at 6 weeks and declined sharply (4.99-fold) at 9 weeks, in opposition to dendrobine biosynthesis. Thus, dendrobine biosynthesis was negatively correlated with *TPS21* expression. *AACT* was the first key enzyme-coding gene in the biosynthesis of sesquiterpenes; the marked stimulation (3.14-fold) of *AACT* at 9 weeks suggested that it might take an active role in the regulation of dendrobine biosynthesis.

By comparing the expression levels of key enzyme-coding genes involved in the MEP pathway between the model group and the control, we found that there were no significant correlations between their expression levels and dendrobine biosynthesis (Supplementary Fig. S5).

### Postulation of the dendrobine biosynthetic pathway

**Figure 11:**
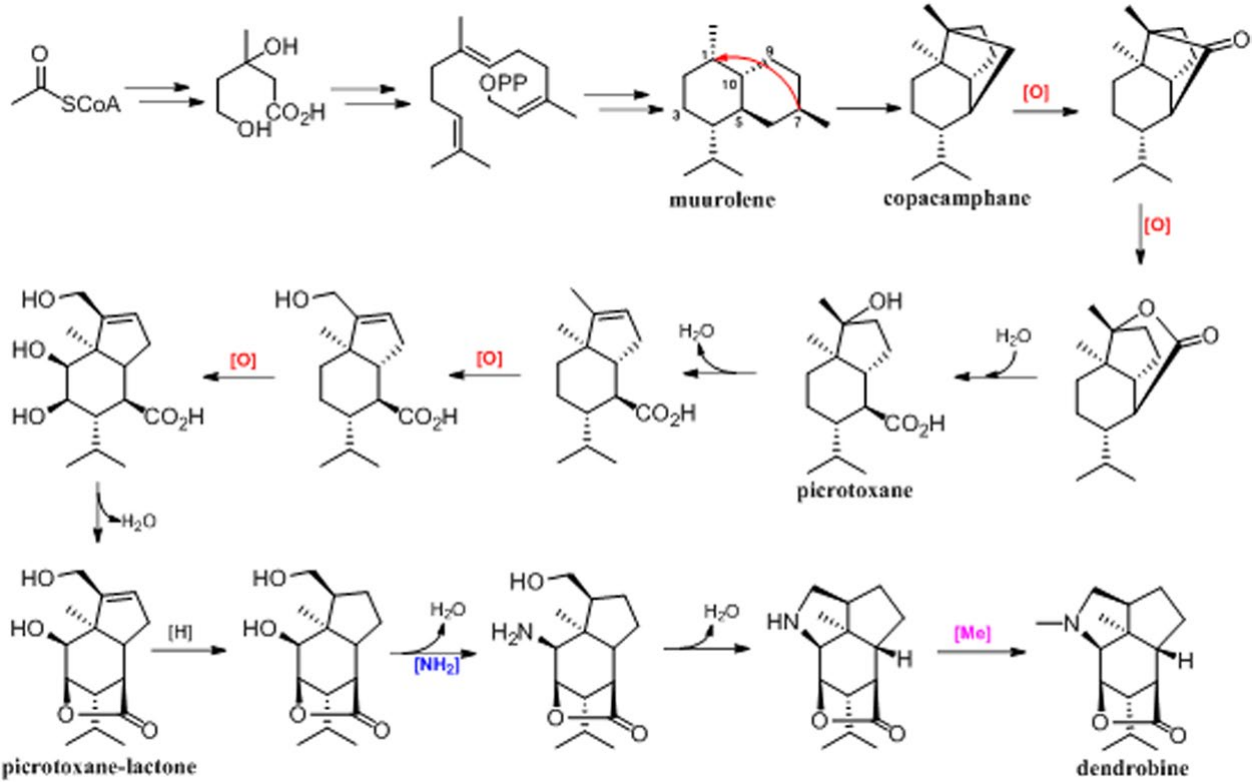
Postulation of the dendrobine biosynthetic pathway.

From the structural features of dendrobine and the sesquiterpene intermediates or precursors isolated from *D. nobile*, the dendrobine biogenetic pathway was suggested systematically (Fig. 11). Acetyl-CoA first forms mevalonate, then shapes the key precursor FPP, which then constructs the skeleton of muurolene-type sesquiterpene. Intra-molecular rearrangement at C-1 and C-7 in muurolene-type sesquiterpene forms copacamphane-type one, from which picrotoxane and pirotoxane-lactone frameworks are established by a series of oxidation reactions. Finally, the modification of amination and methylation cause the formation of dendrobine from the pirotoxane-lactone skeleton. The possible biosynthetic pathway of dendrobine implies that different post-modified enzymes, including oxidase, methyltransferase, and aminotransferase, might play crucial roles in shaping the complex skeleton of dendrobine.

### Differentially expressed post-modification enzymes

The post-modification enzymes involved in the dendrobine biosynthesis pathway mainly included cytochrome P450, aminotransferase, and methyltransferase. By analysing the DEGs, we obtained 49 (cytochrome P450), 13 (aminotransferase) and 49 (methyltransferase) unigenes. Most of them were expressed at high levels, and the ones with top enrichment are listed in Table 2.

**Table 2:** DEGs involved in post-modification enzymes.

Pathway	Gene name	Unigene ID	log2 fold	Padj
Post-modification enzyme	Cytochrome P450 71D55 (*CYP71D55*)	c76734_g1	2.8699	4.45E-10
	Cytochrome P450 735A (*CYP735A*)	c76925_g1	1.6167	0.004607
	Cytochrome P450 71D10 (*CYP71D10*)	c73585_g1	5.3193	0.041079
	Cytochrome P450 94C1 (*CYP94C1*)	c88693_g2	2.6363	2.30E-12
	Methyltransferase-like protein 23 (*METTL23*)	c69161_g1	2.6038	2.19E-07
	Histone-lysine *N*-methyltransferase ATX4 (*ATX4*)	c89731_g2	2.2402	0.021889
	Alanine aminotransferase 2 (*AAT2*)	c88693_g5	2.2991	3.48E-06
	*D*-alanine aminotransferase (*DAT*)	c83743_g5	1.4039	0.018442
	Branched-chain-amino-acid aminotransferase 2 (*BCAT2*)	c82241_g1	2.0736	3.14E-06

### Validation and expression analysis of genes involved in post-modification

**Figure 12:**
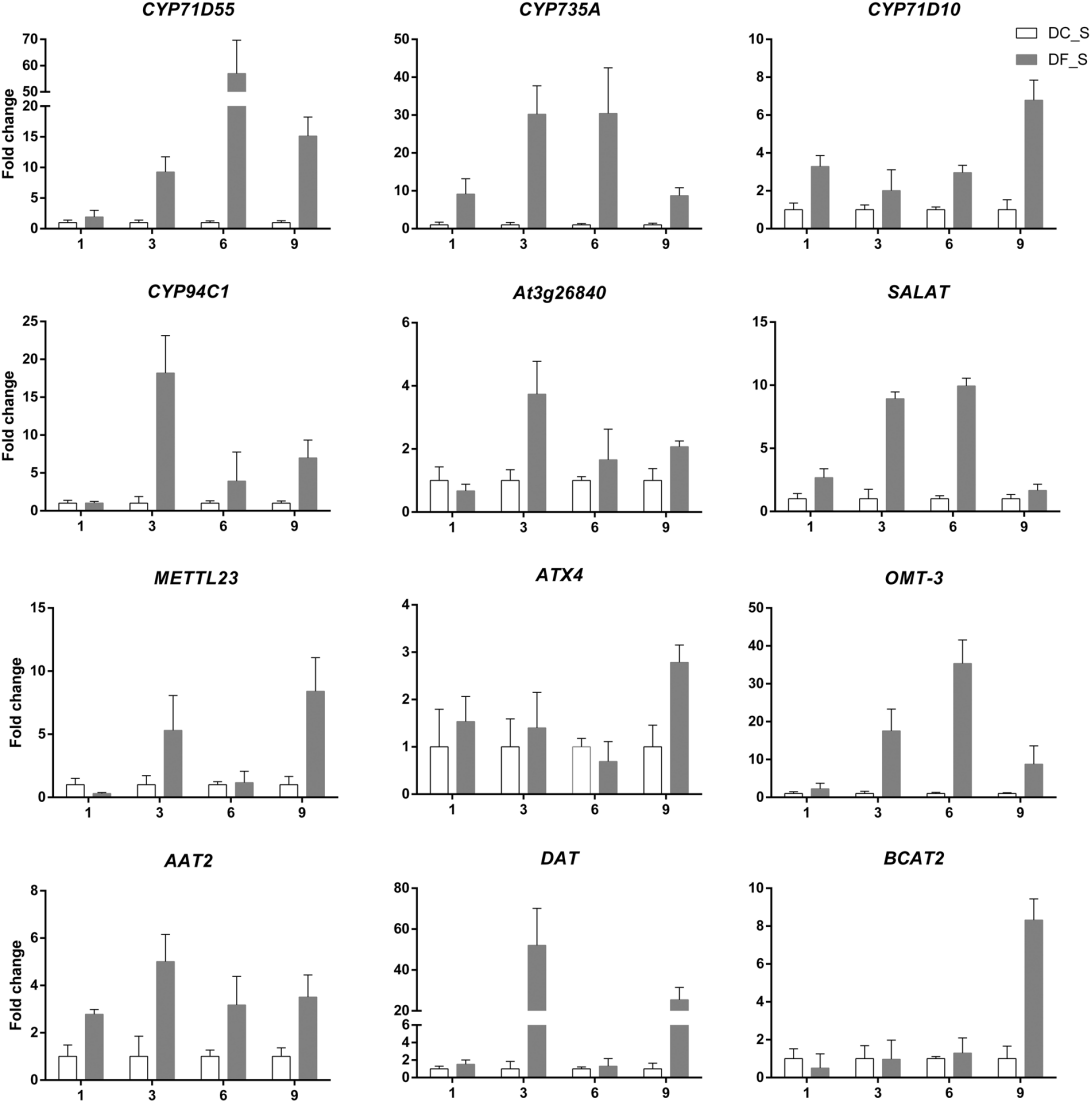
qRT-PCR analysis of key enzyme-coding genes involved in post-modification in *D. nobile*. The y-axes correspond to the mean fold changes of expression values, and the x-axes display the times of symbiotic culture (/weeks). White bars represent the control group (DC_S), and grey bars represent the model group (DF_S). For each qRT-PCR validation, three technical replicates were used, with a minimum of three biological replicates.

The expression levels of 9 genes encoding post-modification enzymes were assessed by qRT-PCR (Fig. 12). The expression levels of all genes were stimulated to different degrees after inoculation. Among them, *CYP1D10*, *METTL23*, *ATX4* and *BCAT2* exhibited higher expression levels at 9 weeks after inoculation than any other stages, implying that these genes might have positive effects on promoting dendrobine biosynthesis. Moreover, the expression of *ATX4*, which encoded *N*-methyltransferase was at a low level at 6 weeks (low content of dendrobine) and was activated at 9 weeks (high content of dendrobine). It was postulated that there was a positive relationship between *ATX4* expression and dendrobine biosynthesis, which might play an important role in the regulation process of dendrobine biosynthesis influenced by MF23.

## Discussion

Early in the 1960s, it was demonstrated that mycorrhizal fungi could offer nutrients, such as glucose, directly to the host^28^. Later research revealed that mycorrhizal fungi could produce some types of phytohormones supplied to their hosts^29^. Recently, with the development of molecular technology, findings of mycorrhizal fungi promoting *Dendrobium* growth or metabolism by regulating gene expression have often been reported^30–32^. Nevertheless, there is little knowledge of the exact mechanism of how mycorrhizal fungi influence their host *Dendrobium*. Driven by the positive impact of mycorrhizal fungi on *Dendrobium* growth and metabolism, we performed morphological, physiological and molecular investigations to obtain substantial knowledge of the interrelationship between mycorrhizal fungus and its host. This report detailed the first possible mechanism for the development of dendrobine content induced by MF23, helping us to understand the relationship between the mycorrhizal fungus and its host.

Peloton, as a sign of the establishment of typical orchid mycorrhizae^33^, is in some ways considered a nutritional pool and nutritional transformation centre to deliver nutrients from the host and environment to those plant cas^34^. In this study, morphological observation of the model and the control groups revealed that at 9 weeks, pelotons were formed together with an increase in the dendrobine content. Since dendrobine biosynthesis requires a great deal of energy and nutrition^35, 36^, these results implied that MF23 might promote dendrobine biosynthesis by forming peloton to supply nutrition to its host plant, *D. nobile*.

Alkaloids are a type of secondary metabolite of plants that play significant roles in defensive responses to environmental stresses. For instance, binary stress induced an increase in indole alkaloid biosynthesis in *Catharanthus roseus* ^37^. Under drought stress, the alkaloid content in the roots of motherwort was increased to a maximum of 1.7 times higher than the control group, with a significant difference (P < 0.01)^38^. When infected by *Ceratocystis fimbriata*, several alkaloids in mango increased at different degrees of resistance to infection^39^. Similarly, increased levels of MF23, as a foreign invader for *D. nobile*, might elicit plant defence, which is likely to include the alkaloid (dendrobine) biosynthetic pathway.

Transcriptome analysis of model and control groups from 1 week revealed that 1388 DEGs were involved in many biological processes, cellular components and molecular functions. Moreover, the effect of MF23 on *D. nobile* was formed at the early stage of symbiotic culture, even before the hyphae encounter the root. These results implied that there might exist some fungal elicitor^40, 41^ or physical contact between MF23 and its host to effect the physiological changes in *D. nobile* to promote dendrobine biosynthesis.

*HMGS* and *HMGR* have been recognized as fairly important key enzymes in the MVA pathway^42, 43^. Many studies provided evidence of a positive correlation between sesquiterpene yield and the expression of these two genes^44, 45^. However, unexpectedly, this report does not indicate a specific relationship between these two genes and the dendrobine content, as shown by RNA-seq and qRT-PCR. These results suggested that the genes might not take an active role in the process of dendrobine biosynthesis infected by MF23.

As the first enzyme of the MVA pathway, AACT condenses two molecules of acetyl-CoA to form acetoacetyl-CoA in terpenoid backbone biosynthesis^46^. PMK and MVD are crucial ATP-dependent enzymes in the MVA pathway that directly affect IPP biosynthesis, the building block of sesquiterpene skeletons^47^. Three genes, *AACT*, *PMK* and *MVD*, encoding the corresponding enzymes were highly expressed with increased in dendrobine content. Previous reports confirmed that product accumulation of downstream reactions in MVA pathway was enhanced in *AACT* over-expressing transgenic plants^48^. Therefore, *AACT* might take a similar role in the dendrobine biosynthesis induced by MF23. In addition, the increases in *PMK* and *MVD* expression levels in the model group improved the dendrobine content, which might be due to increased IPP biosynthesis. In recent years, homologous genes of *PMK* and *MVD* have been cloned from *Amomum villlosum*, *Ginkgo biloba*, and *Eleutherococcus senticosus* ^49–51^. A report proved that *PMK* was highly expressed in the roots of *Aconitum heterophyllum* Wall where the aconite terpenoid alkaloids were synthesized and accumulated^52^. Our results were similar to those in the reports mentioned above, but our work investigated the *PMK* expression level in *D. nobile* for the first time and was the first report on the effect of the *MVD* gene on regulating dendrobine biosynthesis, which provided a foundation for further research of *PMK* and *MVD*.

Sesquiterpene synthases catalyse the formation of the sesquiterpene backbone from FPP^53^. Thus, these enzymes are widely regarded as the rate-determining regulatory steps in MVA pathways. In this study, the expression level of *TPS21*, a gene encoding sesquiterpene synthase, was negatively correlated with dendrobine biosynthesis. According to the structural features of its catalysate humulene (Supplementary Fig. S6), TPS21 was not the specific sesquiterpene synthase involved in dendrobine biosynthesis. Moreover, diverse reports have proved that different types of sesquiterpene synthases may exist in one plant, and these enzymes compete as they consume the common precursor, FPP, to synthesize different types of sesquiterpenes^54^. Therefore, the low *TPS21* expression level might cause more FPP to flux to the biosynthetic pathway of dendrobine, and *TPS21* might affect dendrobine biosynthesis indirectly after plants are infected by MF23.

Post-modification enzymes, such as cytochrome P450, aminotransferase, *N*-methyltransferase were essential for all plant secondary metabolic pathways, including the biosynthesis of alkaloids, hormones, UV protectants, signalling molecules, fatty acids, pigments, defence compounds, *et al.* ^55–58^. It has been confirmed that approximately one-half of the proposed 19 enzymes in the taxol biosynthesis pathway were considered to be cytochrome P450^59^. Different studies revealed that by silencing genes encoding aminotransferases, the biosynthesis of relevant alkaloids in *opium poppy* and *Camellia simensis* could be significantly disrupted^60, 61^. In addition, a report revealed that the expression of genes encoding *N*-methyltransferases was positively correlated with caffeine biosynthesis^62^. According to the postulation of the dendrobine biosynthetic pathway, there are a series of oxidation, amination and methylation reactions in the post-modification process of dendrobine biosynthesis that result in dendrobine formation from the pirotoxane-lactone skeleton. Analysis of transcriptome and qRT-PCR data revealed that the expression levels of genes encoding cytochrome P450, aminotransferase and methyltransferases were up-regulated in the model group, suggesting that MF23 might regulate dendrobine biosynthesis by up-regulating the expression levels of genes encoding these post-modification enzymes. However, since post-modification biological networks are complex and may consist of hundreds of reactions that directly and indirectly intertwine with each other, further research regarding the downstream post-modification processes of dendrobine biosynthesis should be performed.

## Conclusions

By analysing the results of content determination and microscopic observation, we showed the effect of MF23 in increasing the dendrobine content at the early stage of peloton development. To study the molecular mechanism of this process, we generated the first large-scale transcriptome dataset of symbiotic *D. nobile* using RNA-seq. From the dataset, 16 genes involved in the biosynthesis of the backbone of dendrobine sesquiterpenes were identified and validated with qRT-PCR. The significant changes in the expression levels of *AACT*, *MVD*, *PMK* and *TPS21* at 9 weeks after inoculation implied that MF23 might stimulate dendrobine biosynthesis by regulating the expression levels of genes involved in the MVA pathway. In addition, the genes encoding post-modification enzymes, including cytochrome P450, aminotransferase and methyltransferase, were screened for their possible involvement in dendrobine biosynthesis. The results in this report provide a good example for endophytes promoting the formation of bioactive compounds in their host and pave the way for further investigations of the dendrobine biosynthetic pathway.

## Methods

### Biological materials and culture conditions

Tissue culture seedlings of *D. nobile* with 3–5 cm in height were cultured on improved 1/2 Murashige and Skoog (MS) medium, which was supplemented with potato juice (potato 200 g · L^−1^, the juice was boiled for 20 min), 3% (w/v) sucrose, and 0.6% (w/v) agar, pH 5.8. Mycelial plugs from 20-day-old MF23 (*Mycena* sp.) grown on potato dextrose agar medium were placed in the centre of culture bottle, by the side of the roots of *D. nobile*. Then, the culture bottles were kept in a conventional greenhouse with a 10-h light/14-h dark photoperiod at (24 ± 1) °C and an illumination intensity of 1500 Lx. Un-inoculated plants were maintained as a control.

Plant samples were collected at different stages: 1, 3, 6 and 9 weeks post-inoculation (model group) and at the same stages for un-inoculation (control group). All samples were divided into two portions; some samples were used for morphological and chemical research, and the rest were frozen using liquid nitrogen and stored until RNA extraction.

### Growth responses of *D. nobile*

Using one culture bottle of *D. nobile* as a repetition, we maintained a total of 12 repetitions. At each point of time, the maximum height and stem diameter of *D. nobile* were measured. The fresh roots, stems, and leaves from each treatment were weighed and parched at 55 °C. The dry weight was then measured.

### Content determination of dendrobine in *D. nobile*

Stems from each treatment were parched at 55 °C, thoroughly blended and ground to a powder. Standard substances of dendrobine and the internal standard naphthalene were obtained from Sinopharm Chemical Reagent Co, Ltd and Beijing Bei Na Chuang Lian Biotechnology Institute, respectively. The sample preparation for the GC analysis were performed as per the pharmacopoeia of China (2010). Chromatography was performed on an Agilent 6890 GC-FID using an Agilent DB-1 capillary column (0.25 μm × 0.25 mm × 30 m) and nitrogen as a carrier gas.

The experiments were conducted in three replications. Samples were analysed in a random order, with QC samples inserted after every eight samples in the data acquisition sequence. For each sample analysis, 1 μL of derivatized sample was injected. The parameters and the procedures for the GC analysis were performed as per the pharmacopoeia of China (2010). Components of the total ion chromatogram were extracted by the flame ionization detection. Relative correction factors between naphthalene and dendrobine was acquired (f = 1.184849). The linear regression equation y = 0.3135x − 0.022 (r = 0.9994) proved that the dendrobine concentration was linear, with a peak area in the range of 1.1~11 mg · L^−1^. The relative standard deviations (RSDs) of precision, repeatability, stability and recovery rate using this method were 2.1% (n = 5), 2.2% (n = 5), 3.8% (n = 6) and 1.96% (n = 6) (Supplementary Table S4), respectively.

### Light microscopy examination

Fresh root segments were fixed in formalin–acetic acid–alcohol (FAA), an approach described by Feder and O’Brien^63^. Samples were dehydrated in a graded ethanol series, embedded in paraffin, stained with safranine and fast green, sealed with Gel Damar and then observed and photographed on a light microscope equipped with a camera (ZEISS Axio Imager A1).

### RNA extraction, library construction, and sequencing

Total RNA was extracted from 1- and 9-week-old stem samples of *D. nobile* using an RNeasy Plant Mini Kit (cat. nos 74903 and 74904) (Qiagen, Hilden, Germany) following the manufacturer’s instructions. Total RNA degradation and contamination were verified by electrophoresis in a 1.0% agarose gel in 0.5 × TBE (44.5 mM Tris–HCl, 44.5 mM boric acid and 1.25 mM Na_2_EDTA). The concentration of total RNA was checked using a Qubit RNA Assay Kit in a Qubit 2.0 Fluorometer (Life Technologies, Carlsbad, CA, USA). Furthermore, the RNA integrity was assessed using an RNA Nano 6000 Assay Kit with a Bioanalyzer 2100 system (Agilent Technologies, Santa Clara, CA, USA).

A total amount of 1.5 μg RNA per sample was used as the input material for RNA sample preparations. Finally, 8 samples with RNA integrity number (RIN) values above 8 were used for library construction. Sequencing libraries were generated using the NEBNext^®^ Ultra™ RNA Library Prep Kit for Illumina^®^ (NEB, USA) following the manufacturer’s recommendations, and index codes were added to attribute sequences to each sample. Briefly, mRNA was purified from total RNA using poly-T oligo-attached magnetic beads. Fragmentation was performed using divalent cations under elevated temperature in NEBNext First Strand Synthesis Reaction Buffer (5×). First strand cDNA was synthesized using random hexamer primer and M-MuLV Reverse Transcriptase (NaseH^−^). Subsequently, the second strand cDNA synthesis was performed using DNA Polymerase I and RNase H. Remaining overhangs were converted into blunt ends via exonuclease/polymerase activities. After adenylation of 3′ ends of DNA fragments, NEBNext Adaptor with hairpin loop structure was ligated to prepare for hybridization. To select cDNA fragments of preferential 150~200 bp in length, the library fragments were purified with the AMPure XP system (Beckman Coulter, Beverly, USA). Then, 3 μl of USER Enzyme (NEB, USA) was used with size-selected, adaptor-ligated cDNA at 37 °C for 15 min followed by 5 min at 95 °C before PCR. Then, PCR was performed with Phusion High-Fidelity DNA polymerase, Universal PCR primers and Index (X) Primer. Finally, PCR products were purified (AMPure XP system) and library quality was assessed on the Agilent Bioanalyzer 2100 system.

### Sequence reads mapping, assembly, and annotation

Raw data (raw reads) in fastq format were first processed using in-house Perl scripts. In this step, clean data (clean reads) were obtained by removing reads containing adapter, reads containing poly-N and low-quality reads from raw data. At the same time, the Q20, Q30, GC-content and sequence duplication level of the clean data were calculated. All of the downstream analyses were based on clean data with high quality. The left files (read1 files) from all libraries/samples were pooled into one large left.fq file, and right files (read 2 files) were also pooled into one large right.fq file. Transcriptome assembly was performed based on the left.fq and right.fq using Trinity^64^ with min_kmer_cov set to 2 by default and all other parameters set to defaults. Gene function was annotated based on the following seven databases: Nr (NCBInon-redundant proteinsequences), Nt (NCBI non-redundant nucleotide sequences), Pfam (Protein family), KOG/COG (Clusters of Orthologous Groups of proteins), Swiss-Prot (A manually annotated and reviewed protein sequence database), KO (KEGG Ortholog database) and GO (Gene Ontology), using BLAST with a cutoff E-value of 10^−5^.

### Quantification and differential expression analysis of transcripts

Gene expression levels were estimated using RSEM51 for each sample. Clean data were mapped back onto the assembled transcriptome, and the read count for each gene was then obtained from the mapping results. Differential expression analysis of the two groups was performed using the DESeq R package (1.10.1). DESeq52 provides statistical routines for determining differential expression in digital gene expression data using a model based on the negative binomial distribution. The resulting P-values were adjusted using the Benjamini and Hochberg’s approach for controlling the false discovery rate. Genes with an adjusted P-value < 0.05 identified by DESeq were assigned as differentially expressed.

### GO and KEGG enrichment analysis of differentially expressed transcripts

GO enrichment analysis of the DEGs was implemented with the GOseq R packages based on Wallenius non-central hyper-geometric distribution^65^, which can be adjusted for gene length bias in DEGs. KEGG^66, 67^ is a database resource for understanding high-level functions and utilities of the biological system, such as the cell, the organism, and the ecosystem, from molecular-level information, especially from large-scale molecular datasets generated by genome sequencing and other high-throughput experimental technologies (http://www.genome.jp/kegg/). KOBAS^68^ software was used to test the statistical enrichment of DEGs in KEGG pathways.

### Confirmation of the infection-responsive expression profiles by qRT-PCR

DEGs identified using the above described methods were validated with qRT-PCR. The qRT-PCR was performed with the SYBR® Premix ExTaq^TM^ (TaKaRa, Dalian, China) on an ABI 7500 Real-Time PCR System (Applied Biosystems, Foster City, CA, USA). The actin gene of *D. nobile* was used as a reference control^69^. All primers designed for *D. nobile* are shown in Supplementary Table S5. The reaction was performed using the following conditions: denaturation at 95 °C for 30 s, followed by 40 cycles of amplification (95 °C for 5 s, 60 °C for 34 s). For each sample, three technical replicates of the qRT-PCR assay were used with a minimum of three biological replicates. Gene expression was evaluated using the 2^−ΔΔCt^ method^70^.

### Statistical analysis

SPSS 19.0 (IBM, Chicago, IL, USA) statistical software was used for the statistical evaluation of the results. All results are expressed as the means ± standard deviations (SD) of the number of experiments. An unpaired t-test for the values was performed at P < 0.05.

## Electronic supplementary material

Suppkementary material
