# Supplementary material for: Transcriptome Analysis of Genes Involved in Dendrobine Biosynthesis in Dendrobium nobile Lindl. Infected with Mycorrhizal Fungus MF23 (Mycena sp.)
Source: Sci Rep. 2017 Mar 22;7:316. doi: 10.1038/s41598-017-00445-9 (PMC5428410; doi:10.1038/s41598-017-00445-9)
Supplement: Supplementary file 1 — Suppkementary material [file 41598_2017_445_MOESM1_ESM.pdf]

Transcriptome Analysis of Genes Involved in Dendrobine Biosynthesis in *Dendrobium nobile* Lindl. Infected with Mycorrhizal Fungus MF23 (*Mycena* sp.)

Qing Li, Gang Ding, Biao Li* & Shun-Xing Guo*

Institute of Medicinal Plant Development, Peking Union Medical College, Chinese Academy of Medical Sciences, Beijing 100193, People's Republic of China

*Corresponding author: B. L. libiao@126.com ; S. X. G. sxguo1986@163.com

Supplementary Figure S1. The effect of MF23 on growth of *D. nobile* tissue culture seedlings.

Supplementary Figure S2. Length distribution of assembled unigenes.

Supplementary Figure S3. Terpenoid backbone biosynthesis pathway.

Supplementary Figure S4. Sesquiterpenoid and triterpenoid biosynthesis pathway.

Supplementary Figure S5. qRT-PCR analysis of key enzyme-coding genes involved in the MEP pathway in *D. nobile*.

Supplementary Figure S6. The structure of humulene.

Supplementary Table S1. Summary of sequences analysis.

Supplementary Table S2. BLAST analysis of non-redundant unigenes against public databases.

Supplementary Table S3. Unigenes involved in backbone formation of sesquiterpene alkaloid dendrobine biosynthesis.

Supplementary Table S4. Recovery test of dendrobine.

Supplementary Table S5. Primers designed for qRT-PCR.

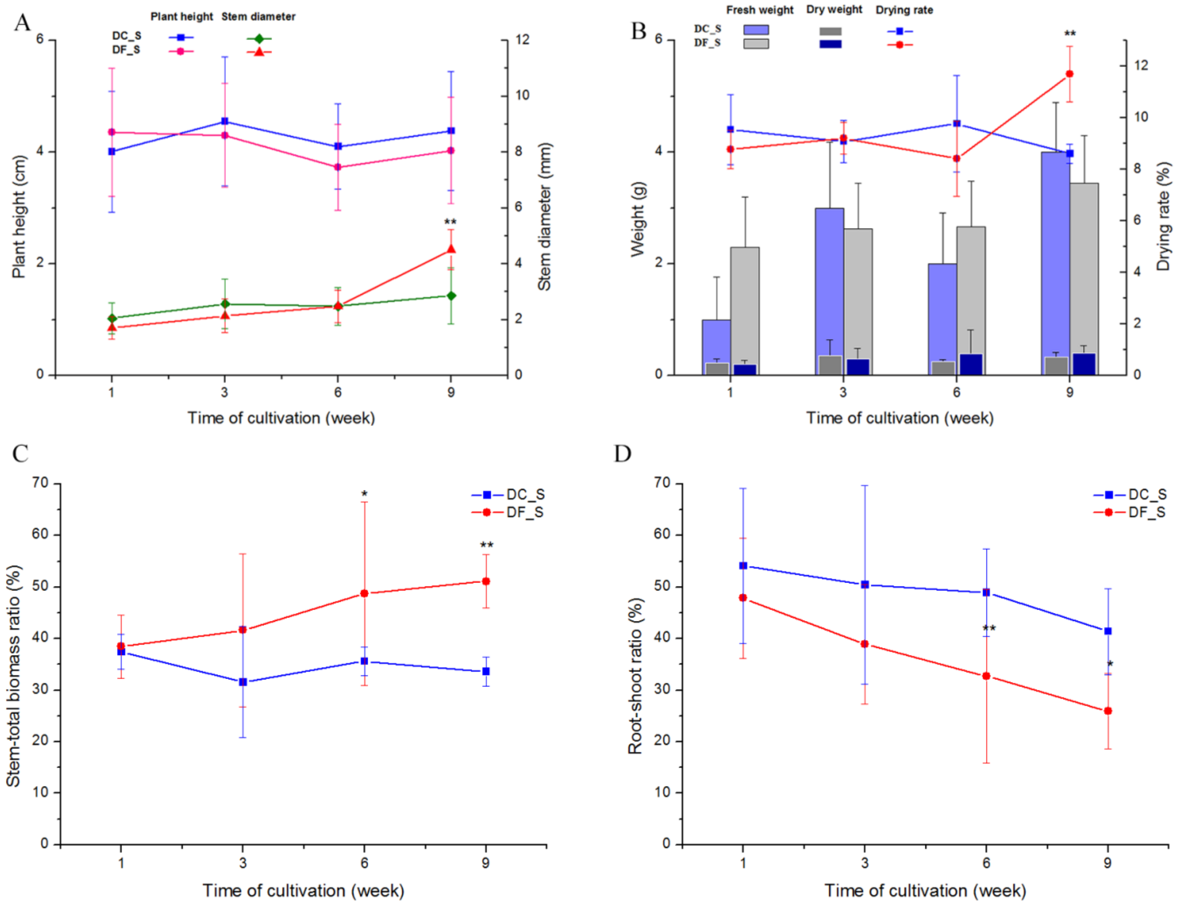

Supplementary Figure S1. The effect of MF23 on the growth of *D. nobile* tissue culture seedlings. (A) Plant height and stem diameter of *D. nobile* tissue culture seedlings. **(B)** Biomass of *D. nobile* tissue culture seedlings. **(C)** Stem-total biomass ratio of *D. nobile* tissue culture seedlings. **(D)** Root-shoot ratio of *D. nobile* tissue culture seedlings. DC_S represents for control group, DF_S represents for model group. Values are presented as the means±SD (n=12); values with * are of statistical significance at $P< 0.05$, values with ** are of statistical significance at $P<0.01$.

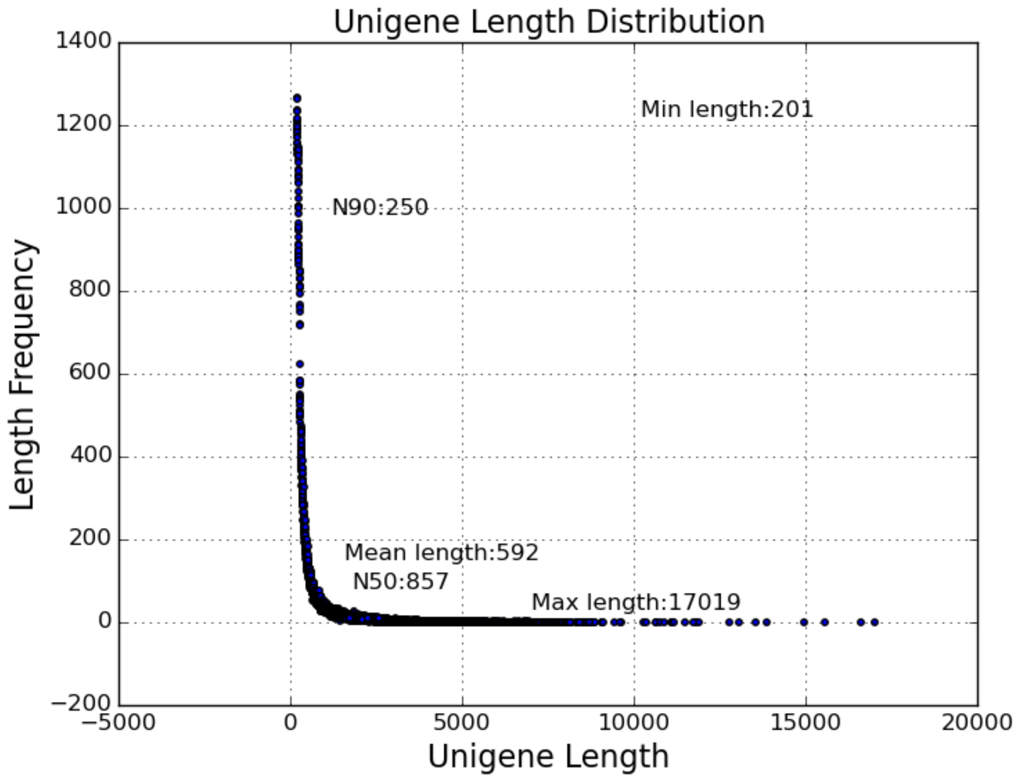

Supplementary Figure S2. Length distribution of assembled unigenes.

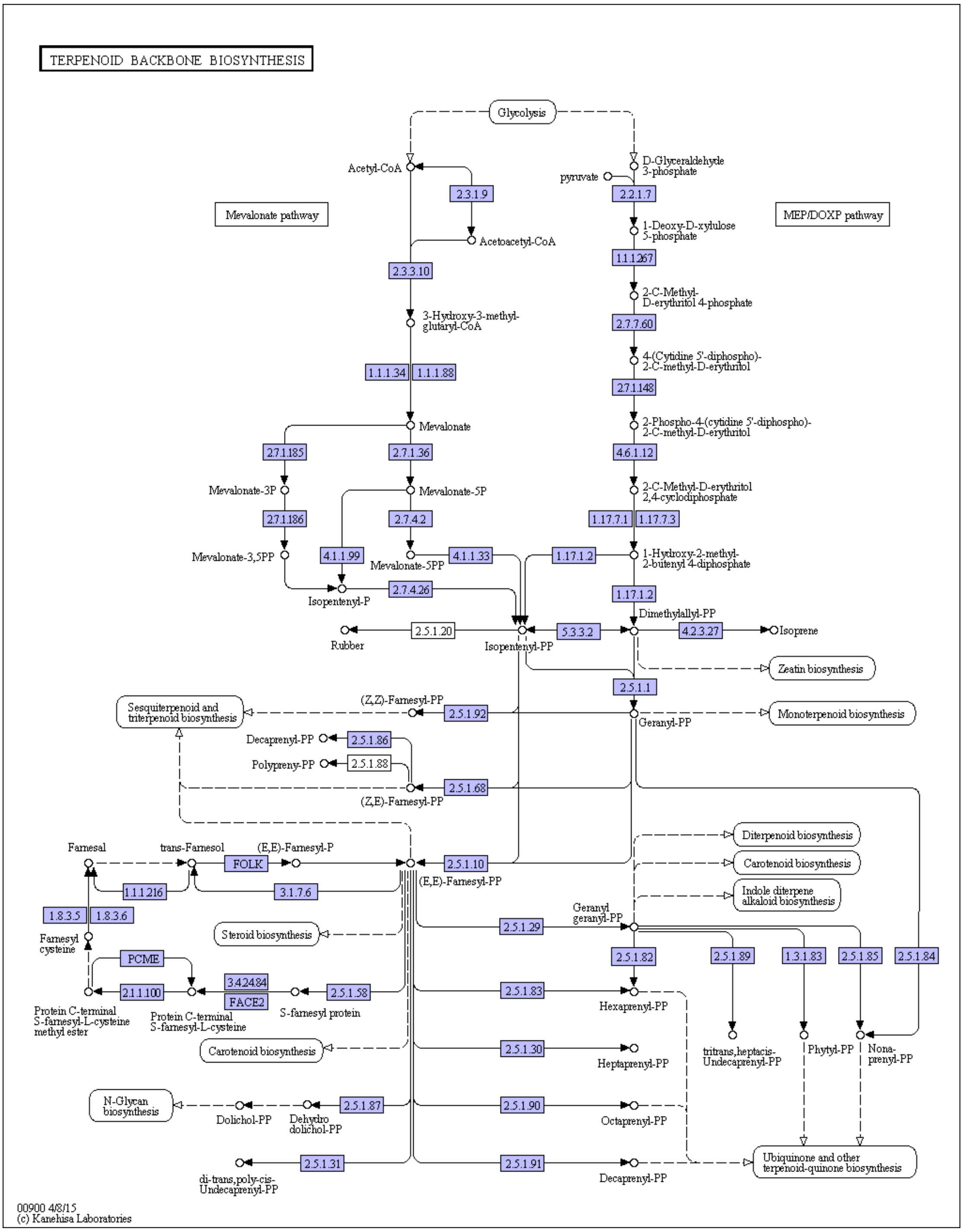

Supplementary Figure S3. Terpenoid backbone biosynthesis pathway (derived from KEGG map00900).

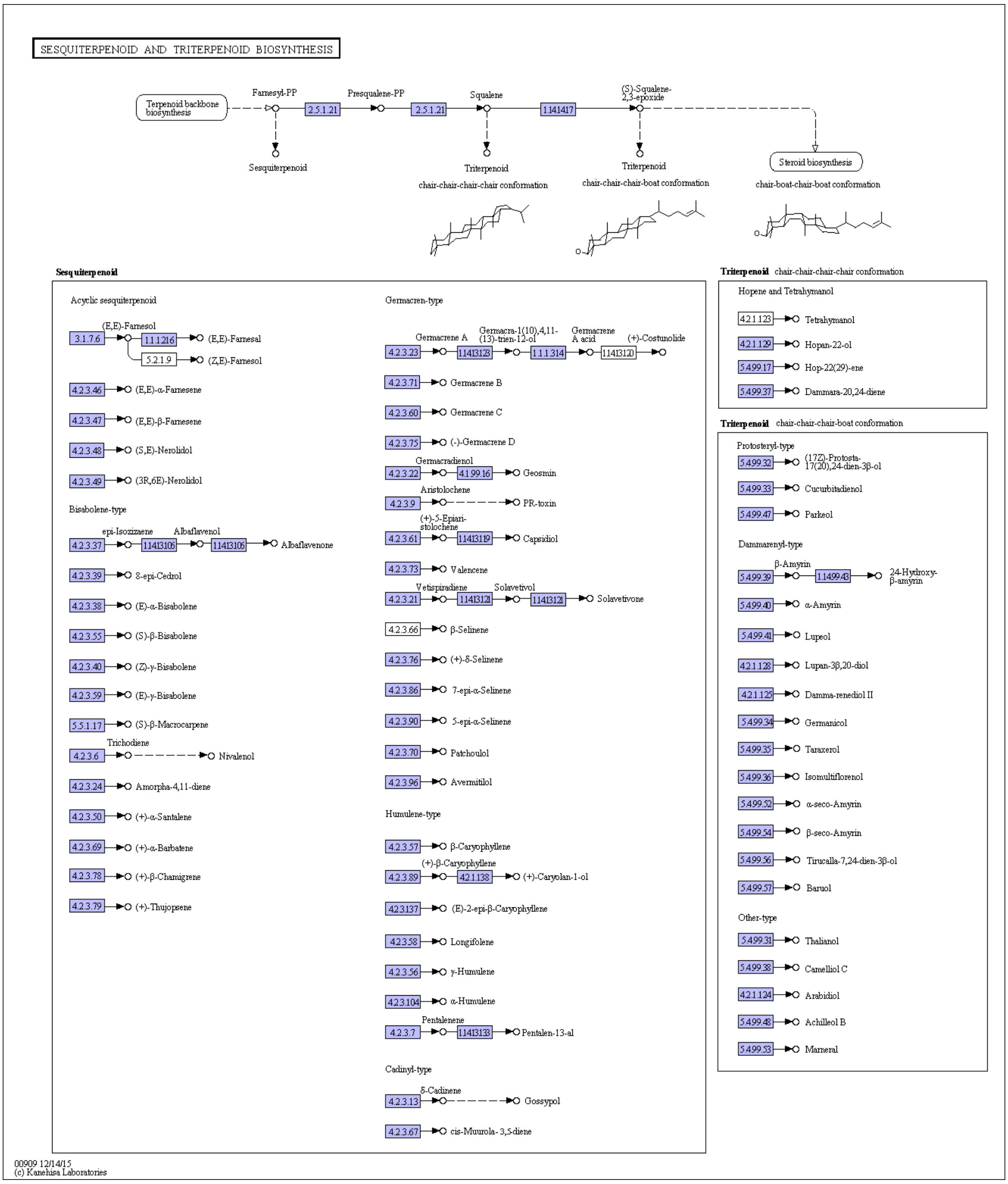

Supplementary Figure S4. Sesquiterpenoid and triterpenoid biosynthesis pathway (derived from KEGG map00909).

.

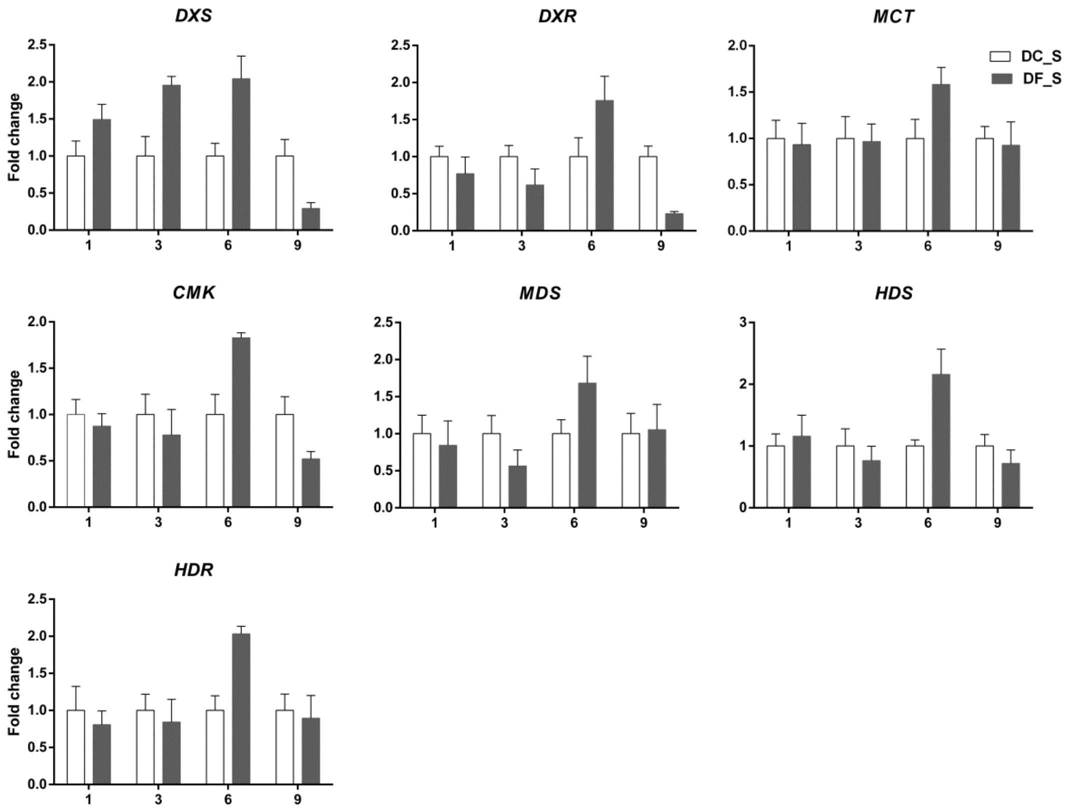

Supplementary Figure S5. qRT-PCR analysis of key enzyme-coding genes involved in the MEP pathway in *D. nobile*. The y-axes correspond to the mean fold changes in expression values, and the x-axes display the times of symbiotic culture (/weeks). White bars represent the control group (DC_S), and grey bars represent the model group (DF_S). For each qRT-PCR validation, three technical replicates were used, with a minimum of three biological replicates.

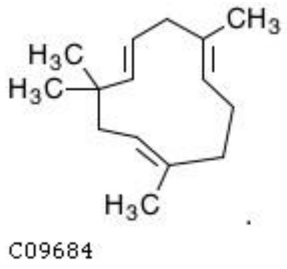

Supplementary Figure S6. The structure of humulene.

Supplementary Table S1. Summary of sequences analysis.

Sample	Raw Reads	Clean Reads	Clean Bases	Error(%)	Q20(%)	Q30(%)	GC Content (%)
DC1_S1	57252114	55277910	8.29G	0.01	97.53	94.05	46.16
DC1_S2	44863528	43312840	6.5G	0.01	97.56	94.09	45.75
DF1_S1	58455694	56340260	8.45G	0.01	97.37	93.7	46.2
DF1_S2	52912596	50959096	7.64G	0.01	97.69	94.33	47.63
DC9_S1	47092876	45414294	6.81G	0.01	97.25	93.47	46.48
DC9_S2	46537374	44964612	6.74G	0.01	97.49	93.96	46.59
DF9_S1	53471134	51541182	7.73G	0.01	97.54	94.09	47.3

DF9_S2	69564340	67080588	10.06G	0.01	97.55	94.12	46.94

Supplementary Table S2. BLAST analysis of non-redundant unigenes against public databases.

Database	Number of Unigenes	Percentage (%)
Annotated in NR	56378	27.19
Annotated in NT	34493	16.64
Annotated in KO	18911	9.12
Annotated in SwissProt	48431	23.36
Annotated in PFAM	46843	22.59
Annotated in GO	47372	22.85
Annotated in KOG	27087	13.06
Annotated in all Databases	8015	3.86
Annotated in at least one Database	83778	40.41
Total Unigenes	207283	100

Supplementary Table S3. Unigenes involved in backbone formation of sesquiterpene alkaloid dendrobine biosynthesis.

Pathway	Gene name	EC
MVA	Acetyl-CoA C-acetyltransferase (*AACT*)	2.3.1.9
	Hydroxymethylglutaryl-CoA synthase (*HMGS*)	2.3.3.10
	3-hydroxy-3-methylglutaryl coenzyme A reductase (*HMGR*)	1.1.1.34
	Mevalonate kinase (*MK*)	2.7.1.36
	Phosphomevalonate kinase (*PMK*)	2.7.4.2
	Diphosphomevalonate decarboxylase (*MVD*),	4.1.1.33
	Geranyl diphosphate synthase (*GPPS*)	2.5.1.1
	Farnesyl pyrophosphate synthetase (*FPPS*)	2.5.1.10
	Alpha-humulene synthase (*TPS21*)	4.2.3.104
MEP	1-deoxy-D-xylulose-5-phosphate synthase (*DXS*)	2.2.1.7
	1-deoxy-D-xylulose-5-phosphate reductoisomerase (*DXR*)	1.1.1.267
	2-C-methyl-D-erythritol 4-phosphate cytidylyltransferase (*MCT*)	2.7.7.60
	4-diphosphocytidyl-2-C-methyl-D-erythritol kinase (*CMK*)	2.7.1.148
	2C-methyl-D-erythritol 2,4-cyclodiphosphate synthase (*MDS*)	4.6.1.12
	4-hydroxy-3-methylbut-2-en-1-yl diphosphate synthase(*HDS*)	1.17.7.3
	4-hydroxy-3-methylbut-2-enyl diphosphate reductase(*HDR*)	1.17.7.4

Supplementary Table S4. Recovery test of dendrobine.

Dendrobine (mg)				Recovery (%)	Average recovery(%)	RSD(%)
Samples level	added	Total	Found			
0.6089	0.5040	1.1129	1.1074	98.9	101.9	1.96
0.6089	0.5040	1.1129	1.1238	102.2		
0.6089	0.5040	1.1129	1.1145	100.3		
0.6089	0.5040	1.1129	1.1237	102.1		

0.6089	0.5040	1.1129	1.1328	104.0
0.6089	0.5040	1.1129	1.1327	104.0

Supplementary Table S5. Primers designed for qRT-PCR.

Genes	Unigene ID	forward primer sequence (5'-3')	reverse primer sequence (5'-3')
AACT	c90222_g1	AGCCTTCCTTGGTTCATTGTCATCT	CTGTGCTGCTATCATTGTTGCCTTC
HMGS	c90508_g3	GCGTTCAGCAGGAATCATTAGAGG	TCTCAAGCAAGGAAGTGACAACTGT
HMGR	c84175_g3	GCCAGCAACATAGTATCAGCCATCT	CACCACCAACAGTTCCTACCTCAAT
MK	c86727_g1	GCCTGCTCAATTCCTGCTATTAACA	GCGAGTTCTGACCTCCATTATTCCT
PMK	c89718_g2	TGGAGGAAGCTATATGCCTGATGTG	CTGCCAGTCGTGTCCGTGTT
MVD	c89821_g1	GGCTACGACGACTGTTGCTGTTAG	CTGAATGCCTCGCTTCTCATCCTC
GPPS	c84183_g4	AACTGGCTCAAGCTGCTGAATACT	ACTGTCGGTTGTGGTATTGGCATAT
FPPS	c79611_g1	TCACTAAGTTCAAGCGTGTCTTCAG	GCTCGTAGCTCGTCCTCTCGTA
TPS21	c91295_g3	GTTGCGGCATCGGAAGGTCATTA	AGGGCGAGATGGAGTGATGGAAA
DXS	c89669_g1	CGCAAAGAGCATCACAAAGCAGATT	GGACCAGGAGCAGGCATAGACT
DXR	c83260_g1	CCAACCTACTCCAACGAACTCACTT	AACTCAATGGACATAGCGGACCTC
MCT	c87740_g2	GCATCCATCGCCGCTATCAATCTA	TCCTTCACTCCAATCCCAACATCTG
CMK	c91297_g3	GGCTGGCAGATGAAGTGGATAAGAA	CGACTGGAACTCCTGGCACATTAG
MDS	c89629_g3	ACGGCGAGGTCACTGCTTCT	AGGCGTCGTTGTTGGCTTGG
HDS	c88411_g1	TCACTGAGCCACCAGAAGAAGAGA	GCAGCAAGAGACCTGTAGAGAAGTT
HDR	c85735_g1	CCGTTCTCTTCTCCGTCACCAT	GCTCCAGCGTCTCCTTCTTGT
CYP71D55	c76734_g1	TGGATGAGGATCTCGTTGATGTTCT	CCGACTGAGCCTTACGCATTATCT
CYP735A	c76925_g1	GAGTGGCAGAAGGCGTTAAGAGA	CGGCGAGTATAGACGAAGAACCT
CYP71D10	c73585_g1	CGAAGCAGCGTGGCAGAGAA	TTGGCGTTGTAGAAGATGGCGTTA
CYP94C1	c88693_g2	CATTCGCCGCAAGTCCTCGTA	GGCTTGACTACCTTCTTCCTCGTTA
At3g26840	c88964_g1	CAACTTCAATGGCTCTCGCATCTG	GCGGCTCTACCACTGTCTCATC
SALAT	c84941_g1	CGCAGTGAGAAGCCAAGTAGAGTT	TCTCCACCTCTTCCTGTCTTCCTTC
METTL23	c69161_g1	AGCGGTCATCATCTGATTGAGTTCT	GCACTTCCTTGGTTCTTCATCTTCA
ATX4	c89731_g2	CCCGAGCCAGTTTCTAAATCCCTTA	AGCCTCCTCTATTGCCAACCTTAAC
OMT-3	c88817_g1	TGACTCTCGCTACCGCCATAGAA	TCGCAGTGGAGCAGGTGAAGA
AAT2	c88693_g5	TCACAGCAATGCGGCGAGTC	CGGCAGAGGAGGAGGAGATGAAT
DAT	c83743_g5	TGAATCGTGCTCCATGTTACCATTG	CCTGCTCGCCTTCTTCTCTTCTC
BCAT2	c82241_g1	GCATCATAGTCGTCGTCATCATCAT	TCCACATCAGCATATTCATCCACAT
